# Factors associated with pneumococcal vaccination in elderly people: a cross-sectional study among elderly club members in Miyakonojo City, Japan

**DOI:** 10.1186/s12889-018-6080-7

**Published:** 2018-10-12

**Authors:** Akihiro Sakamoto, Charnchudhi Chanyasanha, Dusit Sujirarat, Nobuhiro Matsumoto, Masamitsu Nakazato

**Affiliations:** 1 Division of Neurology, Respirology, Endocrinology and Metabolism, Department of Internal Medicine, Faculty of Medicine, University of Miyazaki, Miyazaki, Japan; 2 Faculty of Public Health, Mahidol University, Bangkok, Thailand; 3 Department of Microbiology, Faculty of Public Health, Mahidol University, Bangkok, Thailand; 4 Department of Epidemiology, Faculty of Public Health, Mahidol University, Bangkok, Thailand

**Keywords:** Pneumococcal vaccination, Elderly people, Recommendation, Perception of the severity of pneumonia, Vaccination rate

## Abstract

**Background:**

Pneumonia is the third leading cause of death in Japan. All elderly people aged 65 years or older are recommended to receive a pneumococcal vaccine. A subsidy for part of the cost of routine pneumococcal vaccination in this age group was introduced in 2014. Factors related to vaccination behavior among elderly adults have not been well reported. The purpose of this study was to investigate factors associated with vaccine uptake among elderly people in Japan.

**Methods:**

We conducted a cross-sectional study, using a self-administered questionnaire among elderly club members aged 65 years or older in one city of Japan in April 2017. The participants were selected from among all elderly club members in the study area. Variables extracted from the questionnaire were analyzed using logistic regression analysis.

**Results:**

A total of 208 elderly club members participated in the study. The mean age (± SD) was 77.2 (± 5.3) years. The pneumococcal vaccination rate was 53.2%. Logistic regression analysis revealed three variables that had a significant association with pneumococcal vaccination: a recommendation for vaccination from medical personnel (aOR 8.42, 95% CI 3.59–19.72, *p* <  0.001), receiving influenza vaccination in any of the previous three seasons (aOR 3.94, 95% CI 1.70–9.13, *p* = 0.001), and perception of the severity of pneumonia (aOR 1.23, 95% CI 1.03–1.48, *p* = 0.026).

**Conclusions:**

Although the pneumococcal vaccination rate in this study was increased compared with previous reports, almost half of study participants had not yet received vaccination. Our findings could be helpful for developing vaccination strategies to increase the vaccine coverage in the elderly population.

**Electronic supplementary material:**

The online version of this article (10.1186/s12889-018-6080-7) contains supplementary material, which is available to authorized users.

## Background

Pneumonia is one of the main causes of death worldwide [1]. In Japan, it is the third leading cause of death, following malignant neoplasm and heart disease [2]. The annual incidence of pneumonia in Japanese adults is estimated at 11.9 per 1000 people [3]. Pneumonia can develop in people of all ages; however, it is most commonly found in very young children and people older than 65 years. A recent report from Japan showed that the incidence sharply increased with age, particularly among those over 65 years old [3].

Among causative agents, *Streptococcus pneumoniae*, which is also called pneumococcus, is the most commonly isolated pathogen. Vaccination plays a critical role in prevention; recent studies have supported the effectiveness of pneumococcal vaccine in the prevention of pneumococcal pneumonia or invasive pneumococcal disease [4–10]. There are two types of pneumococcal vaccine that are now available for adults in Japan: 23-valent pneumococcal polysaccharide vaccine (PPV23) and 13-valent pneumococcal conjugate vaccine (PCV13).

In Japan, a national vaccination program for pneumococcal vaccine was not launched until 2014. Before that, the vaccination rate in the elderly population was reported to be 2.6% in 2002 [11], 26.8% in 2008 [12], and 38.3% in 2014 [13]. This was quite low compared with other developed countries; the coverage rate among people aged ≥65 years was 63.5% in the United States [14], 70.1% in the United Kingdom [15], 56.0% in Australia [16], and 57.3% in South Korea [17]. In October 2014, a subsidy program to cover part of the expense for routine pneumococcal vaccination using PPV23 in elderly adults was introduced in Japan. Since the launch of this program, the present vaccine coverage rate is unknown. Furthermore, factors associated with vaccination behavior have not been well reported. The purpose of this study was to investigate factors associated with pneumococcal vaccination among people aged 65 years or older in Japan.

## Methods

### Setting and participants

This was a cross-sectional study using a self-administered questionnaire, conducted in Miyakonojo City located in southwestern Japan. The local population was 162,995 people and that of elderly adults aged ≥65 years was 30.3% in 2017 [18]; this was higher than the national average of 27.6% [19]. In this city, there were 128 elderly clubs. Elderly clubs in Japan are operated on a voluntary basis and organize various activities including health promotion programs. The participants in this study were selected from among all elderly club members. Eligible people were those aged 65 years or older with or without underlying diseases. Individuals who could not understand the contents of the information sheet or the researcher’s explanation were excluded.

### Procedure

Elderly clubs were distributed in 15 districts of the study area, and a monthly meeting was held on a district basis among the members of each elderly club. The researcher attended the monthly meetings in all 15 districts in April 2017 and randomly selected two members from each elderly club. These individuals were invited to participate and recruited into the study.

After the researcher obtained written informed consent, participants completed a survey questionnaire. Questionnaires were collected by the researcher immediately after participants had completed them.

### Instrument

A self-administered questionnaire was developed specifically for this study. The researchers conducted a pre-test among 21 elderly club members in the study area in March 2017, to ensure reliability. Cronbach’s alpha coefficient test was carried out, which resulted in a score higher than 0.70 (0.71 for the knowledge section and 0.76 for the perception section). After that, the questionnaire was finalized.

The questionnaire consisted of five parts, with a total of 44 questions: 1) General characteristics, 2) Predisposing factors, 3) Enabling factors, 4) Reinforcing factors, and 5) Pneumococcal vaccination status. Among these, part 2, 3, and 4 were based on the PRECEDE component of the PRECEDE-PROCEED model, which is a planning model that has been one of the most widely used models for health promotion [20, 21].

1. General characteristics

The questions in this part of the questionnaire addressed age, sex, living status, smoking status, alcohol intake, underlying diseases, and education level.

2) Predisposing factors

This part contained questions regarding knowledge, perception, and health motivation. Knowledge about pneumonia and about pneumococcal vaccine was classified into three levels: high, moderate, and low. Perception was evaluated according to the following four types: perceived susceptibility to pneumonia, perceived severity of pneumonia, perceived benefits of pneumococcal vaccine, and perceived barriers to pneumococcal vaccine. These were based on the components of the Health Belief Model, which is one of the most widely used theories for understanding health behavior [20]. Responses were scored on a five-point Likert scale, with a minimum score of 1 and a maximum of 5, and classified into two levels: higher perception and lower perception. Health motivation was classified into two levels: higher and lower. Details on the classification of knowledge, perception, and health motivation are mentioned in Additional file 1.

3) Enabling factors

This part included questions about past experience of pneumonia and past influenza vaccination status.

4) Reinforcing factors

This part of the questionnaire included questions about recommendations and reminders about the pneumococcal vaccine. Recommendations referred to advice for vaccination received from doctors, nurses, or other health professionals and people such as family members and friends. Reminders referred to information about pneumococcal vaccination seen in posters, the media (for example, TV and newspapers), or other sources.

5) Pneumococcal vaccination status

This part addressed pneumococcal vaccination uptake among participants.

### Data analysis

Because this study aimed to investigate the potential influencing factors for receiving pneumococcal vaccination, vaccination status among participants was considered the dependent variable and all others were treated as independent variables. All variables were analyzed descriptively. Comparison of qualitative variables between groups was performed using a chi-square or Fisher’s exact test. For quantitative variables, a *t*-test was used. Then, the variables with *p* <  0.05 in univariate analysis were included in the binomial logistic regression. A two-tailed *p* < 0.05 was considered statistically significant. The data were analyzed using SPSS version 18.0 (SPSS Inc., Chicago, IL, USA).

### Sample size

Sample size calculation was based on the formula of estimating a finite population proportion: Np(1 − p)Z^2^_1 − α/2_ / d^2^(N − 1) + p(1 − p)Z^2^_1 − α/2_.

There were a total number of 5235 elderly club members in the study area (*N* = 5235). The pneumococcal vaccination coverage rate in Japan among people aged ≥65 years was 38.3% in 2014, according to the Ministry of Health, Labour and Welfare [13]. In October of this year, the subsidy program for pneumococcal vaccination was introduced. Therefore, the coverage rate at the time of this study was estimated to be 45% (*p* = 0.45). The maximum allowable error was set at 15% of this estimated rate (d = 0.0675). The level of statistical significance (α) was 0.05 (Z_1 − α/2_ = 1.96). Consequently, 201 participants was the minimum sample size.

## Results

Elderly club members aged 65 years or older were randomly selected and recruited to this study in April 2017. A total of 208 elderly people participated.

### General characteristics and vaccination status of participants

The descriptive characteristics and vaccination status of participants are shown in Table 1. More than half of respondents were female (60.1%). The mean age (± standard deviation [SD]) of all respondents was 77.2 ± 5.3 years (range, 65–90 years). The group aged 75–84 years accounted for 60.9% of respondents. Most participants (75.1%) lived with others and most (62.1%) had some sort of underlying disease. Among respondents, 53.2% (95% CI 49.8–56.6) had received the pneumococcal vaccine in the past.

**Table 1:** Descriptive characteristics and vaccination status of participants

	n (%)	
Sex (*n* = 208)		
Male	83	(39.9)
Female	125	(60.1)
Age (years old) (*n* = 207)		
65–74	63	(30.4)
75–84	126	(60.9)
85 +	18	(8.7)
Mean ± S.D. (range)	77.2 ± 5.3 (65–90)	
Living status (*n* = 201)		
Living with others	151	(75.1)
Living alone	50	(24.9)
Smoking Status (*n* = 208)		
Current smoker	6	(2.9)
Never smoker	171	(82.2)
Past smoker	31	(14.9)
Alcohol intake (*n* = 203)		
Current drinker	88	(43.3)
Never drinker	111	(54.7)
Past drinker	4	(2.0)
Underlying disease (*n* = 203)		
no Underlying disease	77	(37.9)
with Underlying disease	126	(62.1)
Diabetes Mellitus ^a^	25	(12.3)
Heart disease ^a^	20	(9.9)
Respiratory disease ^a^	16	(7.9)
Renal disease ^a^	7	(3.4)
Immune system disorder ^a^	2	(1.0)
Others ^a^	78	(38.8)
Education level (*n* = 203)		
Primary school	8	(3.9)
Secondary school	100	(49.3)
High school	73	(36.0)
Vocational school	10	(4.9)
College/University or more	12	(5.9)
Vaccination status (*n* = 203)		
Vaccinated	108	(53.2)
Unvaccinated	95	(46.8)

^a^multiple answers possible

### Predisposing factors

The classification of knowledge, perception, and motivation among participants is shown in Table 2. For knowledge, 44.2% of respondents had a high knowledge level about pneumonia, whereas only 24% had a high level of knowledge about pneumococcal vaccine. Regarding participants’ perception, most respondents had a higher level of perceived susceptibility to pneumonia (higher vs. lower: 58.7% vs. 41.3%). In contrast, lower levels predominated for perceived severity of pneumonia (higher vs. lower: 45.7% vs. 54.3%) and for perceived benefits of pneumococcal vaccine (higher vs. lower: 38.9% vs .61.1%). For perceived barriers to pneumococcal vaccine, both levels were quite similar (higher vs. lower: 50.5% vs 49.5%). As for health motivation, 82.0% of respondents were classified as having higher motivation.

**Table 2:** Classification of knowledge, perception, and motivation

	n (%)	
Knowledge level (*n* = 208)		
Knowledge about pneumonia		
High knowledge	92	(44.2)
Moderate knowledge	39	(18.8)
Low knowledge	77	(37.0)
Knowledge about pneumococcal vaccine		
High knowledge	50	(24.0)
Moderate knowledge	42	(20.2)
Low knowledge	116	(55.8)
Perception level (*n* = 208)		
Perceived susceptibility to pneumonia		
Higher perception	122	(58.7)
Lower perception	86	(41.3)
Perceived severity of pneumonia		
Higher perception	95	(45.7)
Lower perception	113	(54.3)
Perceived benefits of pneumococcal vaccine		
Higher perception	81	(38.9)
Lower perception	127	(61.1)
Perceived barriers to pneumococcal vaccine		
Higher perception	105	(50.5)
Lower perception	103	(49.5)
Health motivation level (*n* = 206)		
Higher motivation	169	(82.0)
Lower motivation	37	(18.0)

### Enabling factors and reinforcing factors

Among respondents, 16.1% had developed pneumonia in the past. For influenza vaccination history, 72.1% of participants had received influenza vaccine in any of the previous three seasons. Less than half of respondents had received a recommendation to receive the pneumococcal vaccine from both a medical personnel and family members or friends (38.9% and 39.3%, respectively). In contrast, most participants (90.5%) had obtained information about the pneumococcal vaccine via posters, the media, or other sources (Table 3).

**Table 3:** Distribution of enabling factors and reinforcing factors

	n (%)	
Enabling factors		
Past experience of pneumonia (*n* = 205)	33	(16.1)
Influenza vaccination in any of the previous 3 seasons (*n* = 204)	147	(72.1)
Reinforcing factors		
Recommendation from medical personnel (*n* = 203)	79	(38.9)
Recommendation from familiar people (*n* = 201)	79	(39.3)
Information by poster, media, or other sources (*n* = 201)	182	(90.5)

### Factors associated with pneumococcal vaccination

The following variables were significant in univariate analysis using a chi-square test, Fisher’s exact test, or *t*-test: sex, influenza vaccination in any of the previous three seasons, recommendation for vaccination from medical personnel, vaccine recommendation from family or friends, knowledge about pneumococcal vaccine, and each of the four types of perception (Additional file 2). These variables were further analyzed using logistic regression analysis. An association was maintained for three variables: influenza vaccination in any of the previous three seasons (aOR 3.94, 95% CI 1.70–9.13, *p* = 0.001), recommendation for vaccination from medical personnel (aOR 8.42, 95% CI 3.59–19.72, *p* < 0.001), and perceived severity of pneumonia (aOR 1.23, 95% CI 1.03–1.48, *p* = 0.026) (Table 4).

**Table 4:** Factors associated with pneumococcal vaccination

	Adjusted OR (95% CI)	*p* value
Sex		
Male	1.0	
Female	0.94 (0.44–1.99)	0.871
Influenza vaccination in any of previous 3 seasons		
No	1.0	
Yes	3.94 (1.70–9.13)	0.001
Recommendation from medical personnel		
No	1.0	
Yes	8.42 (3.59–19.72)	< 0.001
Recommendation from familiar people		
No	1.0	
Yes	1.46 (0.67–3.16)	0.339
Knowledge level about pneumococcal vaccine		
Low	1.0	
Moderate	2.40 (0.95–6.06)	0.064
High	1.75 (0.73–4.22)	0.212
Perceived susceptibility to pneumonia		
	0.84 (0.64–1.09)	0.186
Perceived severity of pneumonia		
	1.23 (1.03–1.48)	0.026
Perceived benefits of pneumococcal vaccine		
	0.98 (0.83–1.17)	0.837
Perceived barriers to pneumococcal vaccine		
	1.08 (0.96–1.21)	0.188

## Discussion

In the present study, three variables had a significant association with pneumococcal vaccination: having received a recommendation for vaccination from medical personnel, having received influenza vaccination in any of the previous three seasons, and perceived severity of pneumonia.

Elderly people who had a recommendation for vaccination from medical personnel were approximately 8.4 times more likely to receive a pneumococcal vaccine (aOR 8.42, 95% CI 3.59–19.72, *p* < 0.001); this was the most strongly associated variable. Meanwhile, a recommendation from people such as family members or friends showed no significant association. These findings suggest that medical professionals such as doctors and nurses are considered influential as an advice source by elderly people. Previous studies have also showed the importance of a recommendation from physicians or healthcare providers [22, 23]. Some reports state that the number of physician visits in the previous year is an important factor in receiving pneumococcal vaccination [24–26].

Receiving influenza vaccination at least once in the previous three seasons also had a strong association with pneumococcal vaccination (aOR 3.94, 95% CI 1.70–9.13, *p* = 0.001). An association between influenza vaccination history and pneumococcal vaccination have been reported previously [23, 25, 27, 28]. Vaccination itself is a fundamental and important measure to prevent disease. Therefore, people with awareness about receiving any kind of vaccination are considered to be more concerned about disease prevention and to take a proactive stance regarding pneumococcal vaccination.

Perceived severity of pneumonia was another important motivating factor for pneumococcal vaccination among elderly adults (aOR 1.23, 95% CI 1.03–1.48, *p* = 0.026). Perception of the severity of pneumonia indicates an individual’s belief that, once they develop pneumonia, it could be serious or life threatening. The reason for our finding was presumed to be as follows: participants who had this type of perception held a belief that the consequences of disease could possibly have a great impact not only clinically (death, disability, and pain) but also socially (effects on work, family life, and social relationships); consequently, these people were more likely to receive vaccination. An association between an individual’s perception and pneumococcal vaccination has been reported in some studies [23, 27, 28], supporting our result.

In this study, health motivation level did not show a significant association with pneumococcal vaccination (*p* = 0.088). This was possibly because of the characteristics of the study population. Each elderly club provides various kinds of activities, including health-related ones, such as exercise programs. Thus, elderly club members, even those with lower health motivation in this study’s classification, are considered more health-conscious compared with the general population.

Knowledge is considered an important factor that can lead to positive health-related behavior. However, knowledge level, both about pneumonia and about pneumococcal vaccination, had no association with pneumococcal vaccination in this study. This could have occurred because knowledge is more or less related to an individual’s perception. As a result, these two variables conceivably confounded each other, and no association was found for knowledge level.

The pneumococcal vaccination rate was 53.2% in this study. This was much higher than the rates in previous reports from Japan: 2.6% in 2002 [11], 26.8% in 2008 [12], and 38.3% in 2014 [13]. The predominant reason for this increase was likely because of the introduction of the subsidy program. Presently, elderly people aged 65 years or older (and people aged ≥60 years and < 65 years with specific medical conditions) are eligible for subsidized routine pneumococcal vaccination. Every fiscal year, around 20% of the eligible elderly population (aged 65, 70, 75, 80, 85, 90, 95, and 100 years) are assigned to the subsidy program. Since the introduction of this subsidy in October 2014, 2.5 years had passed and nearly half of elderly adults aged ≥65 years had been assigned to the subsidy by the time of this study. Therefore, it was expected that an increased number of elderly people have been vaccinated owing to the subsidy. In some countries, the introduction of a national immunization program or universal funding has been successful in increasing the pneumococcal vaccine coverage in the elderly population [16, 17, 24]. One study from Japan also supports this point, reporting that the pneumococcal vaccination rate was 52.1% in municipalities where the full cost was subsidized by the free vaccination campaign for victims of the 2011 Great East Japan Earthquake [29]. Thus, the introduction of the subsidy is considered to have played an important role in the increased vaccination rate in our study.

We found improvement in study participants’ awareness about the pneumococcal vaccine. In Japan, an individual’s recognition toward the pneumococcal vaccine was previously quite low. One study reported that only 18% of patients over 60 years old who had a chronic respiratory disease knew of the existence of the pneumococcal vaccine [11]. For the past several years, information about the pneumococcal vaccine and its subsidy has been disseminated in the media, posters, pamphlets, and so on. In the present study, 90.5% of respondents stated that they had obtained information about pneumococcal vaccine from a poster, the media, or other sources.

### Limitations

Our study has several limitations. First, the study population comprised Japanese elderly club members; therefore, the results are not generalizable to the entire elderly population. Second, pneumococcal vaccination status was self-reported using a questionnaire and was not verified by medical records; thus, this information may be subject to recall bias. Third, this was a cross-sectional study; therefore, we could not establish causality. Hence, there are likely other factors affecting pneumococcal vaccination behavior that were not accounted for in this study.

## Conclusion

Our study revealed that the vaccination rate of the pneumococcal vaccine was 53.2%. Although the vaccination rate was increased compared with previous reports, almost half of study participants had not yet received vaccination. Three variables, recommendation for vaccination from medical personnel, receiving influenza vaccination in any of the previous three seasons, and perceived severity of pneumonia, had significant associations with pneumococcal vaccination behavior among the elderly adults in this study.

Elderly clubs in Japan organize various kinds of activities including health education. Therefore, they are a suitable potential provider of a proposed health promotion program to improve the rate of pneumococcal vaccination. Our study revealed the importance of advice from medical personnel. This health promotion program provided by the elderly club, by providing workshops or seminars with medical personnel, is expected to be an effective measure for encouraging vaccination. Furthermore, this may be a good opportunity to remind older people of the seriousness of pneumonia and its consequences, which was another influencing factor for pneumococcal vaccination in our study.

## Additional files

Additional file 1: Classification of knowledge, perception, and health motivation. (PDF 100 kb)

Additional file 2: Univariate analysis. (PDF 168 kb)

## Abbreviations

aOR: Adjusted odds ratio
CI: Confidence interval
PCV: Pneumococcal conjugate vaccine
PPV: Pneumococcal polysaccharide vaccine
SD: Standard deviation

## References

[1] World Health Organization. The top 10 causes of death. 2017. http://www.who.int/mediacentre/factsheets/fs310/en/. Accessed 5 June 2018.

[2] Ministry of Health, Labour and Welfare. Vital statistics. 2016. http://www.mhlw.go.jp/toukei/saikin/hw/jinkou/geppo/nengai16/dl/gaikyou28.pdf. Accessed 5 June 2018.

[3] Morimoto K, Suzuki M, Ishifuji T, Yaegashi M, Asoh N, Hamashige N (2015). The burden and etiology of community-onset pneumonia in the aging Japanese population: a multicenter prospective study. PLoS One.

[4] Moberley S, Holden J, Tatham DP, Andrews RM (2013). Vaccines for preventing pneumococcal infection in adults. Cochrane Database Syst Rev.

[5] Kraicer-Melamed H, O'Donnell S, Quach C (2016). The effectiveness of pneumococcal polysaccharide vaccine 23 (PPV23) in the general population of 50 years of age and older: a systematic review and meta-analysis. Vaccine.

[6] Falkenhorst G, Remschmidt C, Harder T, Hummers-Pradier E, Wichmann O, Bogdan C (2017). Effectiveness of the 23-valent pneumococcal polysaccharide vaccine (PPV23) against pneumococcal disease in the elderly: systematic review and meta-analysis. PLoS One.

[7] Bonten MJ, Huijts SM, Bolkenbaas M, Webber C, Patterson S, Gault S (2015). Polysaccharide conjugate vaccine against pneumococcal pneumonia in adults. N Engl J Med.

[8] Tsai YH, Hsieh MJ, Chang CJ, Wen YW, Hu HC, Chao YN (2015). The 23-valent pneumococcal polysaccharide vaccine is effective in elderly adults over 75 years old--Taiwan's PPV vaccination program. Vaccine.

[9] Diao WQ, Shen N, Yu PX, Liu BB, He B (2016). Efficacy of 23-valent pneumococcal polysaccharide vaccine in preventing community-acquired pneumonia among immunocompetent adults: a systematic review and meta-analysis of randomized trials. Vaccine.

[10] Suzuki M, Dhoubhadel BG, Katoh S, Ariyoshi K, Morimoto K (2017). 23-valent pneumococcal polysaccharide vaccine against pneumococcal pneumonia. Lancet Infect Dis.

[11] Watanuki Y, Takahashi H, Yoshiike Y, Ogura T, Sato M, Miyazawa N (2005). Understanding of pneumococcal vaccination in patients with chronic respiratory diseases. Nihon Kokyuki Gakkai zasshi.

[12] Kondo M, Yamamura M, Hoshi SL, Okubo I. Demand for pneumococcal vaccination under subsidy program for the elderly in Japan. BMC Health Serv Res. 2012. 10.1186/1472-6963-12-313. 10.1186/1472-6963-12-313 PMC3470958 22970727

[13] Ministry of Health, Labour and Welfare. Number of vaccinated persons. 2016. http://www.mhlw.go.jp/topics/bcg/other/5.html. Accessed 5 June 2018.

[14] National health interview survey, United States, 2014-2015. 2016. https://www.cdc.gov/nchs/data/nhis/earlyrelease/earlyrelease201605.pdf. Accessed 5 June 2018.

[15] Public Health England. Health protection report. 2016. https://www.gov.uk/government/uploads/system/uploads/attachment_data/file/540290/hpr2416_ppv.pdf. Accessed 5 June 2018.

[16] Dyda A, Karki S, Hayen A, MacIntyre CR, Menzies R, Banks E (2016). Influenza and pneumococcal vaccination in Australian adults: a systematic review of coverage and factors associated with uptake. BMC Infect Dis.

[17] Yang TU, Kim E, Park YJ, Kim D, Kwon YH, Shin JK (2016). Successful introduction of an underutilized elderly pneumococcal vaccine in a national immunization program by integrating the pre-existing public health infrastructure. Vaccine.

[18] Population estimates in Miyazaki prefecture. 2017. http://www.pref.miyazaki.lg.jp/tokeichosa/kense/toke/kako2.html. Accessed 5 June 2018.

[19] Statistic Bureau, Ministry of Internal Affairs and Communications. Population estimates by age (5-year age group) and sex. 2017. http://www.stat.go.jp/data/jinsui/pdf/201712.pdf. Accessed 5 June 2018.

[20] Rimer B, Glanz K. Theory at a glance: a guide for health promotion practice. 2nd ed. Bethesda: National Cancer Institute; 2005.

[21] Green LW, Kreuter MW (2005). Health program planning: an educational and ecological approach.

[22] Dominguez A, Soldevila N, Toledo D, Godoy P, Castilla J, Force L (2016). Factors associated with influenza vaccination of hospitalized elderly patients in Spain. PLoS One.

[23] Ridda I, Motbey C, Lam L, Lindley IR, McIntyre PB, Macintyre CR (2008). Factors associated with pneumococcal immunisation among hospitalised elderly persons: a survey of patient's perception, attitude, and knowledge. Vaccine.

[24] Lu PJ, Nuorti JP (2010). Pneumococcal polysaccharide vaccination among adults aged 65 years and older, U.S., 1989-2008. Am J Prev Med.

[25] Dominguez A, Soldevila N, Toledo D, Godoy P, Torner N, Force L (2016). Factors associated with pneumococcal polysaccharide vaccination of the elderly in Spain: a cross-sectional study. Hum Vaccin Immunother.

[26] Al-Sukhni W, Avarino P, McArthur MA, McGeer A (2008). Impact of public vaccination programs on adult vaccination rates: two examples from Ontario, Canada. Vaccine.

[27] Klett-Tammen CJ, Krause G, Seefeld L, Ott JJ (2016). Determinants of tetanus, pneumococcal and influenza vaccination in the elderly: a representative cross-sectional study on knowledge, attitude and practice (KAP). BMC Public Health.

[28] Liu S, Xu E, Liu Y, Xu Y, Wang J, Du J (2014). Factors associated with pneumococcal vaccination among an urban elderly population in China. Hum Vaccin Immunother.

[29] Naito T, Matsuda N, Tanei M, Watanabe Y, Watanabe A (2014). Relationship between public subsidies and vaccination rates with the 23-valent pneumococcal vaccine in elderly persons, including the influence of the free vaccination campaign after the great East Japan earthquake. J Infect Chemother.

