# Supplementary material for: Factors associated with pneumococcal vaccination in elderly people: a cross-sectional study among elderly club members in Miyakonojo City, Japan
Source: BMC Public Health. 2018 Oct 12;18:1172. doi: 10.1186/s12889-018-6080-7 (PMC6186036; doi:10.1186/s12889-018-6080-7)
Supplement: Supplementary file 1 — Classification of knowledge, perception, and health motivation. (PDF 100 kb) [file 12889_2018_6080_MOESM1_ESM.pdf]

Additional file 1: Classification of knowledge, perception, and health motivation

Knowledge

There are a total of 12 questions for knowledge in the questionnaire: 6 questions for knowledge about pneumonia and 6 for pneumococcal vaccine. They were three-way questions; the answers were “True”, “False”, or “Not sure”. A score of 1 was assigned to correct answer and 0 was given to the rest two (score range: 0–6). Then, knowledge level, both about pneumonia and about pneumococcal vaccine, was classified into three levels as follows.

		**Score**
**High knowledge**	≥80% of the total score	0–3
**Moderate knowledge**	≥60% and <80% of the total score	4
**Low knowledge**	<60% of the total score	5,6

Perception

A total of 16 questions was given to evaluate individual’s perception: perceived susceptibility to pneumonia (3 questions), perceived severity of pneumonia (4 questions), perceived benefits of the pneumococcal vaccine (4 questions), and perceived barriers to pneumococcal vaccine (5 questions). They were scored on a five-point Likert scale. The scoring of the items regarding perceived susceptibility, perceived severity, and perceived benefits was as follows: strongly agree: 5, agree: 4, neither agree nor disagree: 3, disagree: 2, and strongly disagree: 1. Meanwhile, the items on perceived barriers were reversely scored as follows: strongly agree: 1, agree: 2, neither agree nor disagree: 3, disagree: 4, and strongly disagree: 5. The score more than or equal to the mean was classified into higher perception and less than the mean was into lower perception.

**Higher perception**	≥ the mean score
**Lower perception**	< the mean score

mean score in perceived susceptibility to pneumonia = 3.63
mean score in perceived severity of pneumonia = 4.05
mean score in perceived benefits of the pneumococcal vaccine = 4.04
mean score in perceived barriers to pneumococcal vaccine = 3.01

Health motivation

Three questions were given for health motivation. Answers were alternative, “Yes” or “No”. A score

of 1 was given for each “Yes” answer and 0 for “No” answer. Health motivation level was classified into 2 levels: higher motivation and lower motivation. When answered “Yes” for all three questions, it is categorized into higher motivation. The other answering patterns were categorized into lower motivation.

**Higher motivation**	answered “Yes” for all three questions
**Lower motivation**	the others
