# Supplementary material for: Factors associated with pneumococcal vaccination in elderly people: a cross-sectional study among elderly club members in Miyakonojo City, Japan
Source: BMC Public Health. 2018 Oct 12;18:1172. doi: 10.1186/s12889-018-6080-7 (PMC6186036; doi:10.1186/s12889-018-6080-7)
Supplement: Supplementary file 2 — Univariate analysis. (PDF 168 kb) [file 12889_2018_6080_MOESM2_ESM.pdf]

Additional file 2: Univariate analysis

General characteristics	Vaccinated n (%)		Unvaccinated n (%)		p value
**Sex** (n = 202)					0.011
Male	52	(64.2)	29	(35.8)	
Female	56	(45.9)	66	(54.1)	
**Age (years old)** (n = 203)					0.644
65 - 74	31	(49.2)	32	(50.8)	
75 - 84	69	(56.1)	54	(43.9)	
85 +	8	(50.0)	8	(50.0)	
**Living status** (n = 197)					0.965
Living with others	78	(52.7)	70	(47.3)	
Living alone	26	(53.1)	23	(46.9)	
**Smoking Status** (n = 203)					0.782
Current smoker	4	(66.7)	2	(33.3)	
Never smoker	87	(52.4)	79	(47.6)	
Past smoker	17	(54.8)	14	(45.2)	
**Alcohol intake** (n = 198)					0.289
Current drinker	51	(59.3)	35	(40.7)	
Never drinker	52	(48.1)	56	(51.9)	
Past drinker	2	(50.0)	2	(50.0)	
**Underlying disease** (n = 198)					0.121
no Underlying disease	36	(47.4)	40	(52.6)	
with Underlying disease	69	(56.6)	53	(43.4)	
**Education level** (n = 198)					0.775
Primary school	3	(42.9)	4	(57.1)	
Secondary school	52	(53.1)	46	(46.9)	
High school	38	(53.5)	22	(46.5)	
Vocational school	4	(40.0)	6	(60.0)	
College/University or more	8	(66.7)	4	(33.3)	
**Household income** (n = 184)					0.410
0 - 150,000 JPY	32	(48.5)	34	(51.5)	
150,001 - 250,000 JPY	44	(55.7)	35	(44.3)	
250,001 JPY and over	24	(61.5)	15	(38.5)	

**Knowledge level**	**Vaccinated**		**Unvaccinated**		**p value**
	**n (%)**		**n (%)**		
**Knowledge about pneumonia** (n = 203)					0.136
High	56	(60.9)	36	(39.1)	
Moderate	18	(46.2)	21	(53.8)	
Low	34	(47.2)	38	(52.8)	
**Knowledge about pneumococcal vaccine** (n = 203)					0.001
High	35	(70.0)	15	(30.0)	
Moderate	27	(64.3)	15	(35.7)	
Low	46	(41.4)	65	(58.6)	

**Perception**	**Vaccinated**	**Unvaccinated**	**t-test**	**p value**
	**Mean ± SD**	**Mean ± SD**		
**Perceived susceptibility to pneumonia** (n = 203)				
	3.72 ± 0.57	3.53 ± 0.61	2.29	0.023
**Perceived severity of pneumonia** (n = 203)				
	4.20 ± 0.59	3.89 ± 0.72	3.28	0.001
**Perceived benefits of pneumococcal vaccine** (n = 203)				
	4.13 ± 0.57	3.94 ± 0.69	2.05	0.042
**Perceived barrier to pneumococcal vaccine** (n = 203)				
	3.20 ± 0.64	2.99 ± 0.67	2.37	0.019

**Health motivation level**	**Vaccinated**		**Unvaccinated**		**p value**
	**n (%)**		**n (%)**		
**Higher motivation** (n = 203)	93	(56.0)	73	(44.0)	0.088
**Lower motivation** (n = 203)	15	(40.5)	22	(59.5)	

Enabling factors	Vaccinated n (%)		Unvaccinated n (%)		p value
**Past experience of pneumonia** (n = 202)					
Yes	19	(59.4)	13	(40.6)	0.429
No	88	(51.8)	82	(48.2)	
**Influenza vaccination in any of the previous 3 seasons** (n = 201)					
Yes	91	(62.8)	54	(37.2)	< 0.001
No	16	(28.6)	40	(71.4)	

Reinforcing factors	Vaccinated n (%)		Unvaccinated n (%)		p value
**Recommendation from medical personnel** (n = 200)					
Yes	63	(80.8)	15	(19.2)	< 0.001
No	44	(36.1)	78	(63.9)	
**Recommendation from familiar people** (n = 198)					
Yes	56	(71.8)	22	(28.2)	< 0.001
No	49	(40.8)	71	(59.2)	
**Information by poster, media, or others** (n = 198)					
Yes	97	(53.6)	84	(46.4)	0.606
No	8	(47.1)	9	(52.9)	
